# Syncytial nerve net in a ctenophore adds insights on the evolution of nervous systems

**DOI:** 10.1126/science.ade5645

**Published:** 2023-04-20

**Authors:** Pawel Burkhardt, Jeffrey Colgren, Astrid Medhus, Leonid Digel, Benjamin Naumann, Joan J Soto-Àngel, Eva-Lena Nordmann, Maria Y Sachkova, Maike Kittelmann

**Affiliations:** 1 Michael Sars Centre, University of Bergen, 5008 Bergen, Norway; 2 Institut für Biowissenschaften, Allgemeine und Spezielle Zoologie, Universität Rostock, 18055 Rostock, Germany; 3 Oxford Brookes University, Department of Biological and Medical Sciences, Oxford, OX3 0BP, UK

## Abstract

A fundamental breakthrough in neurobiology has been the formulation of the neuron doctrine by Santiago Ramón y Cajal, stating the nervous system is composed of discrete cells. Electron microscopy later confirmed the doctrine and allowed the identification of synaptic connections. Here we used volume electron microscopy and 3D reconstructions to characterize the nerve net of a ctenophore, marine invertebrate belonging to one of the earliest-branching animal lineages. We found that neurons in the subepithelial nerve net have a continuous plasma membrane forming a syncytium. Our findings suggest fundamental differences of nerve net architectures between ctenophores and cnidarians/bilaterians and offer an alternative perspective on neural network organization and neurotransmission.

## The enigmatic nervous system of ctenophores

**Figure 1:**
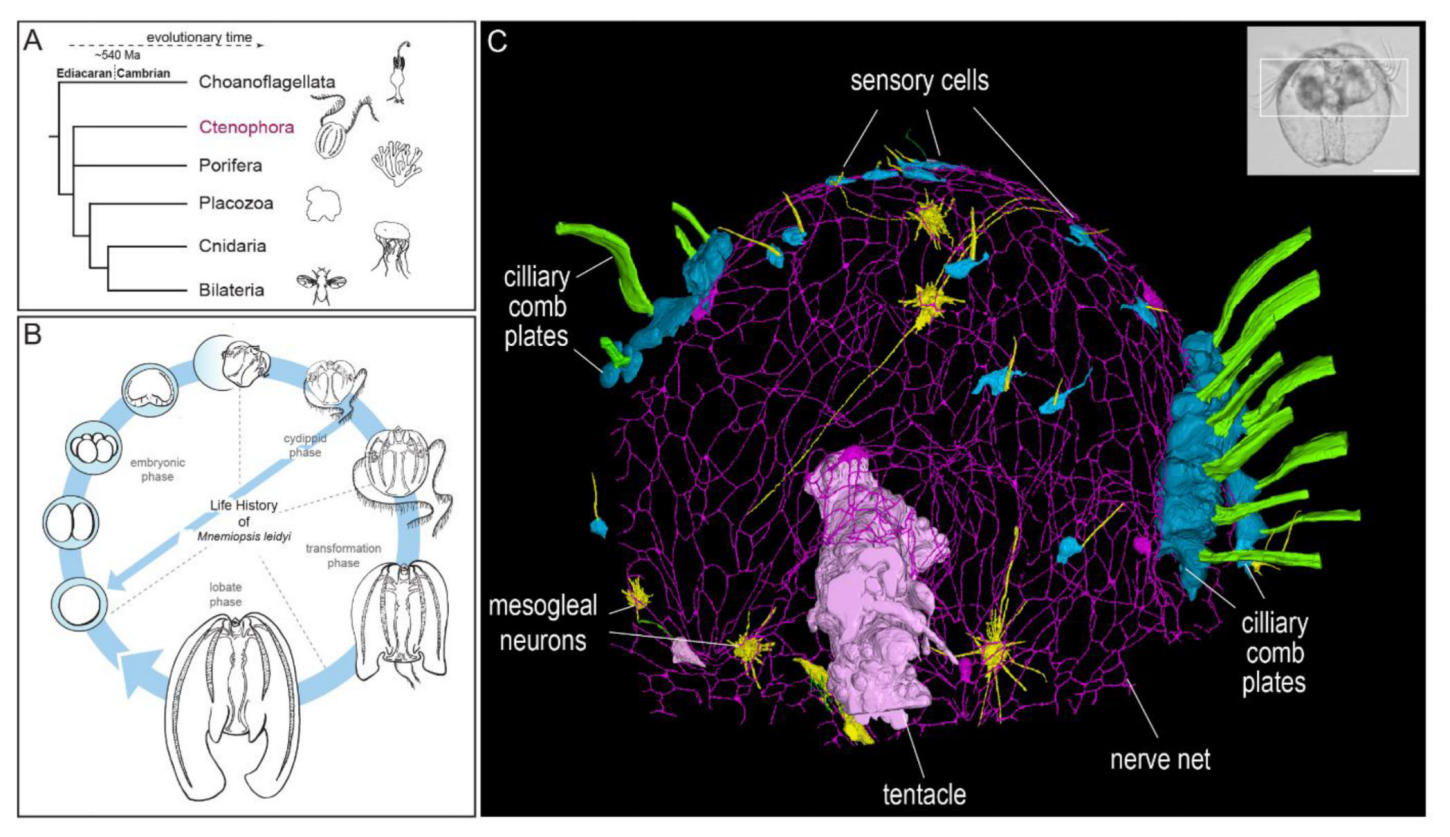
Ctenophores and their nervous system. **(A)** Ctenophores as one of the earliest branching extant lineages of the animal kingdom. **(B)** The ctenophore *Mnemiopsis leidyi* exhibits complex life cycle stages including a predatory cydippid phase that hatches from the egg and can reproduce after a few days. **(C)** 3D reconstruction of the nerve net, comb rows, sensory cells, mesogleal neurons and a tentacle from SBFSEM data of a 1-day old cydippid. Inset: Phase contrast image of a 1-day old cydippid. White box: area reconstructed in C. Scale bar: 100 μm.

For more than one century, the structure and evolutionary origin of the animal nervous system has been at the centre of much debate among biologists. Fundamental progress in our structural understanding was put forward by Santiago Ramón y Cajal, postulating that the nervous system is composed of discrete cells, so-called neurons, rather than forming a syncytial continuum, as proposed by Camillo Golgi(1). The discovery of synaptic connections between individual neurons by electron microscopy later confirmed Cajal’s theory. But is this always the case? There is accumulating evidence that ctenophores, gelatinous marine invertebrates moving through the water column by ciliary comb rows, are among the earliest branching extant lineages of the animal kingdom (Fig. 1A)(2–5). Most ctenophore life cycles include a predatory cydippid stage which, for some species is already able to reproduce a few days after hatching (Fig. 1B)(6). Ancestral state reconstruction suggests the cydippid body plan is a plesiomorphic character of ctenophores(7).

The early split of ctenophores from other groups indicates that a nervous system, and maybe even neurons, could have evolved at least twice – once within the ctenophores and once within the lineage of the remaining animals(8). Initiated by genomic analyses(2, 3), molecular and physiological features of the ctenophore nervous system were subsequently interpreted to support this scenario(4, 5). In contrast to sponges and placozoans, ctenophores exhibit an elaborate nervous system consisting of a subepithelial nerve net (SNN), mesogleal neurons, a sensory aboral organ, tentacle nerves and diverse sensory cells in all parts of their body (Fig. 1C and movie S1)(9–14). Deciphering the development, structure and function of the ctenophore nervous system is a key element to understand the origin and evolution of animal nervous systems. We have recently shown that a large repertoire of lineage-specific neuropeptides has evolved in the ctenophore *Mnemiopsis leidyi*(14). Furthermore, we identified a unique feature of SNN neurons: the multiple neurites extending from one soma are interconnected through anastomoses and thus form an extensive continuous network within a single nerve net neuron(14). This characteristic sets them apart from other animal neurons. Additionally, there was little evidence on how these nerve net neurons connect each other, to sensory neurons and to cells within the mesoglea due to the lack of synaptic markers suitable for fluorescent labeling or large-scale electron microscopic data spanning multiple neurons. Here we used high pressure freezing fixation techniques in combination with Serial Block Face Scanning Electron Microscopy (SBFSEM) to establish the first ultrastructural 3D network of SNN neurons and other cell types in a ctenophore.

## The cydippid SNN is organized in a syncytium

**Figure 2:**
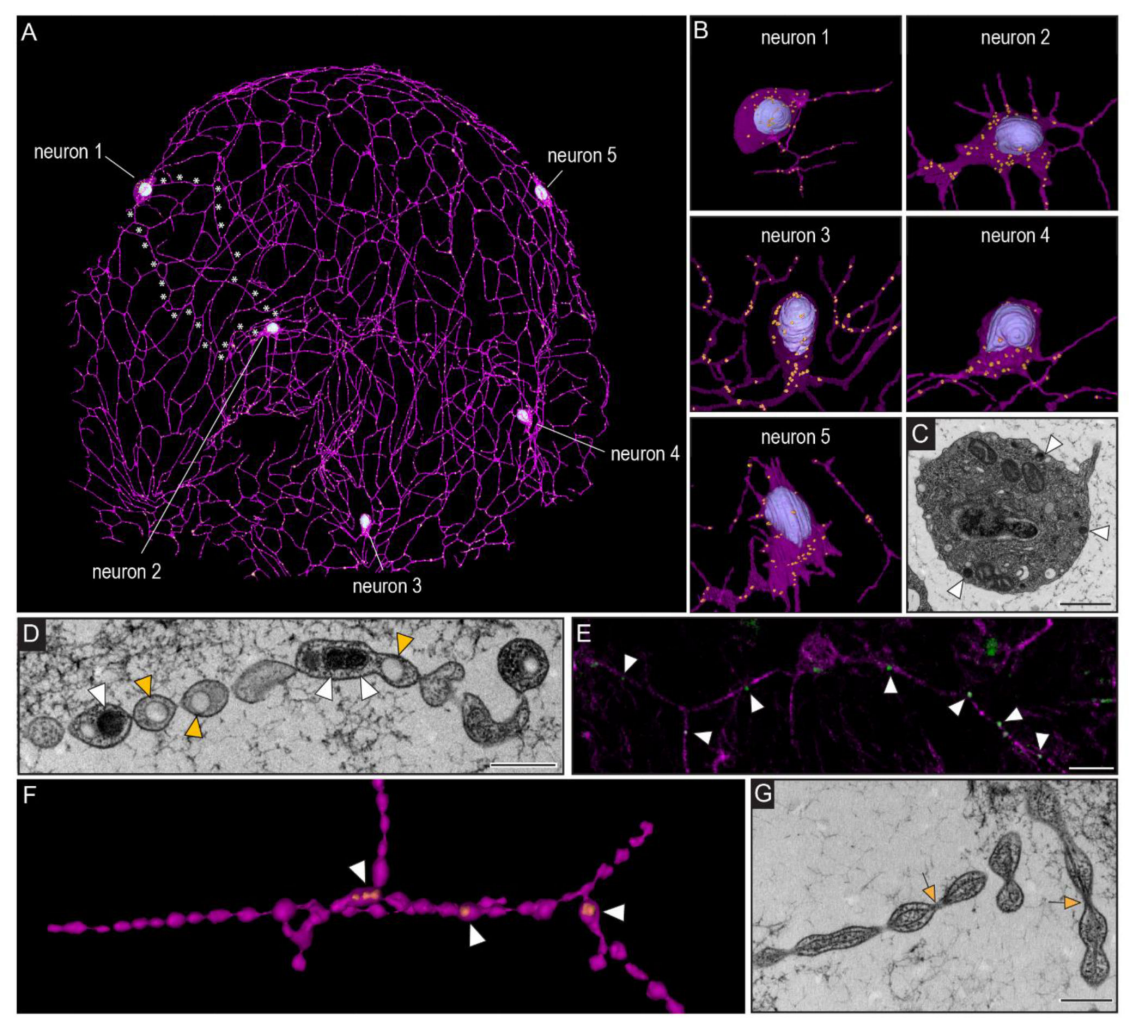
Connectivity and ultrastructure of the ctenophore SNN. **(A)** 3D reconstruction of five SNN neurons. White asterisks indicate examples of continues membrane between cell bodies of neuron 1 and 2. **(B)** 3D reconstruction of the SNN neuron cell bodies showing the nucleus (blue) and dense core vesicles (orange). **(C)** TEM cross section of an SNN neuron cell body showing ultrastructural details including large dense core vesicles (white arrowhead). **(D)** TEM cross section of a SNN neurite with dense core and clear core vesicles localized in “blebbed” areas (white and orange arrowheads). **(E)** Antibody staining against neuropeptide ML02736a (green) in SNN neurites (magenta) stained with anti-tubulin. **(F)** TEM 3D reconstruction of SNN neurite (violet) and dense core vesicles (orange) highlighting the blebbed morphology. **(G)** TEM cross section of SNN neurites showing continuous microtubules (orange arrows) passing through narrow segments. Scale bars C: 1 μm; D, G: 500 nm.

Recent 3D reconstruction of a nerve net neuron in a cydippid-phase *Mnemiopsis leidyi* has revealed a wide network of anastomosed neurites extending from only one soma(14). However, to understand the nature of connections between multiple nerve net neurons as well as other cell types we collected a larger continuous SBFSEM dataset of an early cydippid that includes 5 nerve net neurons, 6 mesogleal neurons and 22 putative sensory cells. The neurites of all five SNN cells were connected through an anastomosed continuous network (Fig. 2A). Whereas gap junctions could readily be identified within comb plates (fig. S1) as previously reported (15), neither electrical nor chemical synapses were detected between the cells of the SNN. This observation was confirmed in smaller datasets of the nerve net beneath two comb rows and along the gut in two other cydippid individuals (fig. S2). Additionally, injection of the fluorescent lipophilic dye 1,1′-Dioctadecyl-3,3,3′,3′-Tetramethylindocarbocyanine Perchlorate (DiI) into only one of the cells of 2-cell staged embryos led to fluorescent signal in only one half of the cydippid body, and the signal was seen in SNN cell bodies throughout the animal consistent with the syncytial nature of the SNN (fig. S3).

Morphologically, neurites within the SNN exhibited no obvious polarity (axon vs. dendrite), showing similar diameter, dense core vesicles distribution throughout their length and the lack of the typical presynaptic triads (Fig. 2A-C). Moreover, SNN neurites often showed a blebbed or “pearls-on-a-string” morphology (Fig. 2D-G and fig. S4). The narrow segments were often just wide enough for microtubules to pass (Fig. 2G, fig. S4), and bulged segments often contained larger clear or electron dense vesicles and occasionally endoplasmic reticulum (Fig. 2D and fig. S4). A recently developed antibody against the neuropeptide ML02736a(14) confirmed the presence of neuropeptides within some of the vesicles of SNN neurons (Fig. 2E, fig. S5). Although SNN neurons seemed to lack synapses between each other, we identified chemical synapses from the SNN to polster cells (fig. S6), suggesting directional signal transmission from the SNN to effector cells.

## Mesogleal neurons form direct contacts with the syncytial SNN

**Figure 3:**
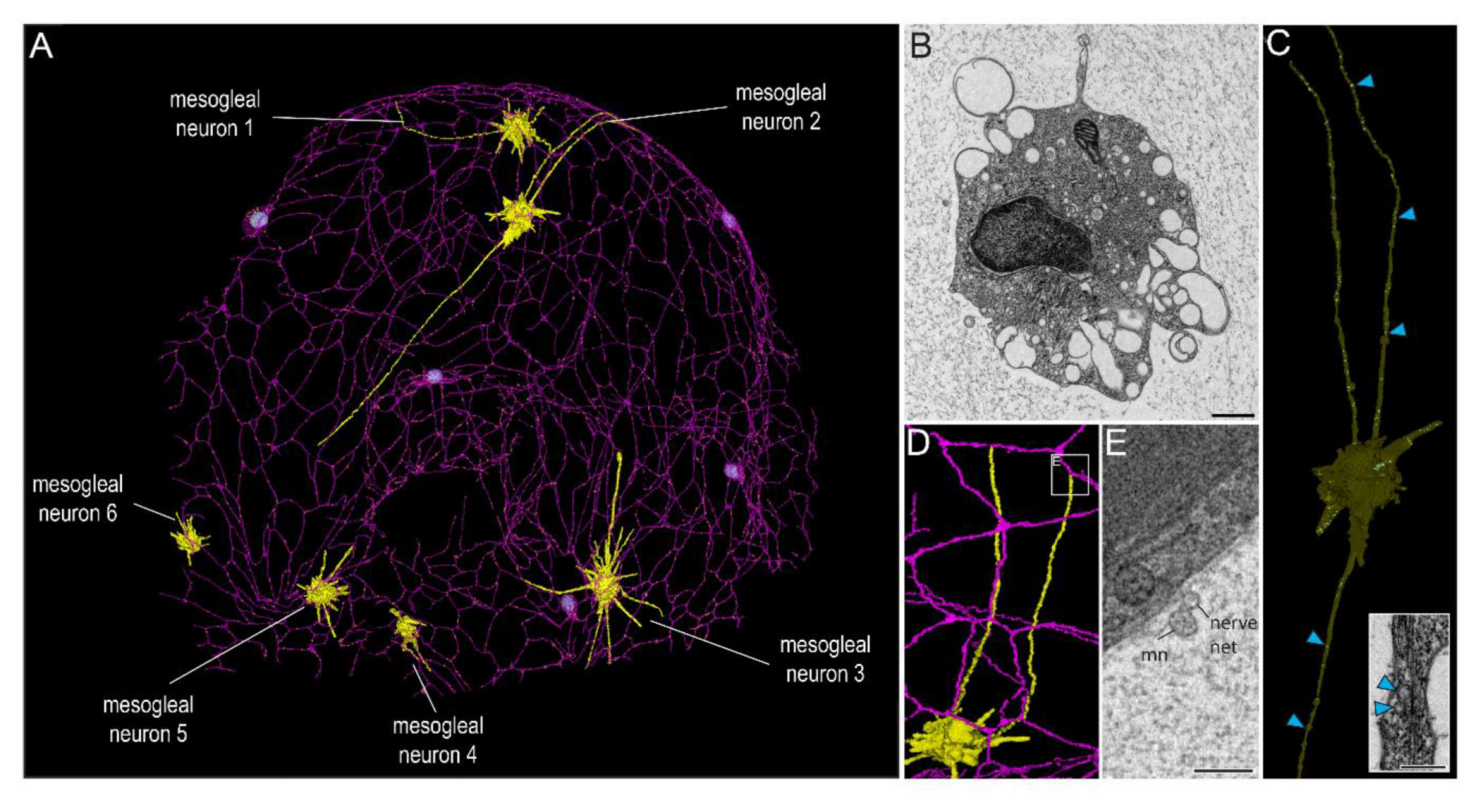
Close association of mesogleal neurons and the SNN. **(A)** 3D reconstruction of SNN (violet) and mesogleal neurons (yellow) from SBFSEM data. **(B)** TEM cross section of a mesogleal neuron cell body. Different types of clear vesicles and vacuoles but no dense core vesicles are present. **(C)** 3D reconstructed mesogleal neuron with three long neurites that contain small clear vesicles (blue arrowheads). TEM cross section of mesogleal neurites with small clear vesicles shown in inset. **(D)** 3D reconstruction of mesogleal neuron with contact site (white box) to SNN. **(E)** Corresponding SBFSEM image of contact site between mesogleal neuron and SNN neuron. mn: mesogleal neuron. No chemical or electric synapse structures could be observed. Scale bars B: 1 μm; C (inset): 200 nm; E: 500 nm.

We identified and reconstructed six mesogleal neurons exhibiting a star-like morphology with extensive plasma membrane protrusions of variable lengths (Fig. 3A). Their somata were filled with a variety of vesicles and larger vacuoles (Fig. 3B) and the protrusions of these cells did not show the “pearls-on-a-strings” morphology present in neurites of the SNN. Some of the protrusions formed plasma membrane juxtapositions to neurites of the SNN (Fig. 3A, D, E). However, we did not find ultrastructural evidence for electrical or chemical synapses (Fig. 3E). In contrast to SNN neurons, we did not observe any electron dense vesicles in mesogleal neurons (Fig. 3B) but instead small electron-lucent vesicles of a similar size as synaptic vesicles (Fig. 3C) suggesting a different type of information transmission.

## Sensory cells form simple circuits involving the syncytial SNN

**Figure 4:**
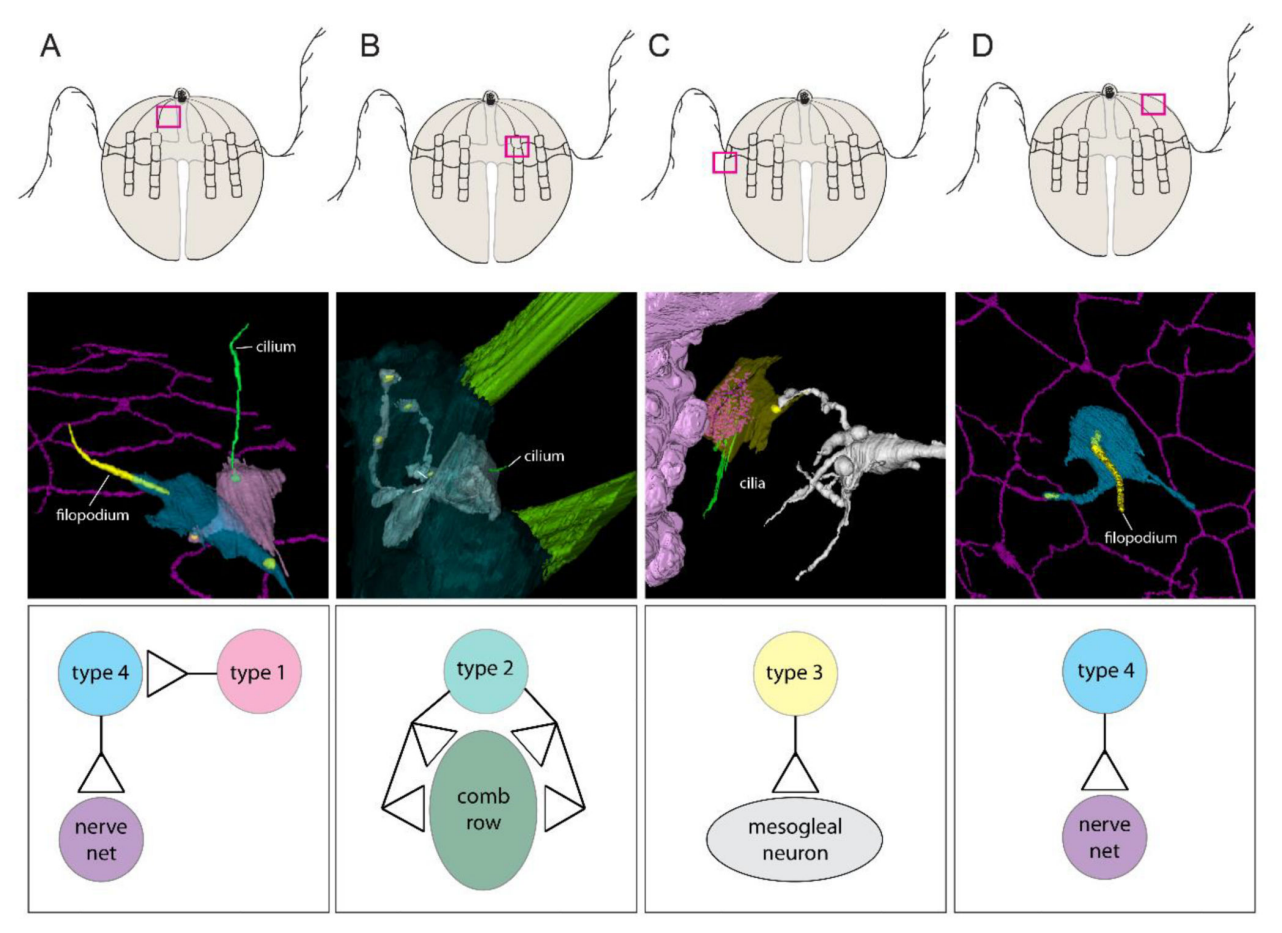
3D reconstruction of sensory cells allows for the identification of simple circuits. Top panel: Localization of each circuit (pink square). Middle panel: 3D reconstructions of sensory and effector cells. Mitochondria are shown in yellow as representative of synaptic tripartite complexes in all circuits. Bottom panel: Proposed wiring diagram. **(A)** Circuit between type 1 and type 4 sensory cell and SNN. **(B)** Multiple synaptic connections between type 2 sensory cell with short cilium and comb cells. **(C)** Synaptic connection between type 3 sensory cell near tentacle and a mesogleal neuron. **(D)** Type 4 sensory cell with single filopodium synapses onto nerve net.

We identified and reconstructed a total of 22 putative sensory cells from the present and an earlier data set(14) which fit into five morphological groupings (Fig. 4, fig. S7 and table S1). Some of them resembled known ctenophore sensory cell types (type 1, 4 and 5)(16, 17) whereas others exhibited a morphology that, at the best of our knowledge, has not been described previously (type 2 and 3) (Fig. 4, fig. S7, and table S1). We detected chemical synapses in several but not all putative sensory cells contacting neuronal or other effector cells (Fig. 4, fig. S7). Type 1 sensory cells exhibited a single long cilium and onion root basal body (Fig. 4, fig. S7A and B). Type 2 sensory cells exhibited a very short single cilium without an onion root basal body. Long neurites extending from their somata formed chemical synapses to polster cells (Fig. 4B, fig. S7A and C).

Type 3 sensory cells exhibited multiple cilia without onion root basal bodies. Many large electron dense vesicles are localized beneath the cilia (Fig. 4C and fig. S7A and D). We found one of these cells near the tentacle with a synaptic connection to a mesogleal neuron (Fig. 4C). Type 4 sensory cells exhibited a single long filopodium. Some of them formed synapses to neurites of the SNN (Fig. 4A and D) and some also received synaptic input from type 1 sensory cells (Fig. 4A). Type 5 sensory cells exhibited multiple long filopodia. They formed plasma membrane contact to polster cells, but we did not detect synaptic contacts from or to this cell type. Finally, we used the 3D ultrastructural evidence to identify several discrete and simple neural circuits in early cydippid-phase *M. leidyi*. These circuits included synaptic signal transmission from sensory cells to other cell types including SNN neurons, mesogleal neurons, polster cells or even other sensory cell types (Fig. 4A-D).

## Discussion

In the debate about the organization of animal nervous system at the end of the 19^th^ century Joseph von Gerlach (1871)(18) and Camillo Golgi (1885)(19) put forward the “reticular theory” (also syncytial theory). Both proposed the cellular continuity of neurons. This view was challenged by Ramón y Cajal (1888)(1) proposing an organization from discrete cellular units connected via synapses. Both contestant theories were founded on Golgi’s newly invented black staining that enabled scientists to study the detailed morphology of neurons and their neurites(20). Golgi and Cajal were honored with the Nobel Prize in Physiology or Medicine in 1906 for their effort in elucidating the architecture of the nervous system(20). However, with the advent of electron microscopy in the 1950s and the discovery of the synaptic cleft, the reticular theory was put to rest in favor of Cajal’s hypothesis(21, 22). In the present study, volume electron microscopy revealed the 3D ultrastructural architecture of the SNN in an early cydippid-phase ctenophore providing evidence for its reticular – or syncytial – organization. Previous work suggested anastomosed nerve cords in adult ctenophores based on chemical staining(9) and multiple parallel strands of anti-tyrosylated-a-tubulin-stained neurites(10). Here we showed that a syncytial nerve net already exists in cydippid-phase *M. leidyi*. This syncytium may be reinforced in adult animals through the anastomosis of additionally formed neurites; however, confirmation of such connectivity will require further detailed high resolution analysis of the nerve net throughout development.

Using high pressure freezing and freeze substitution techniques to preserve fine ultrastructural details with minimal fixation artifacts, we showed that the SNN forms a continuous structure. This is further supported by the unrestricted spread of DiI throughout the nerve net.

Whereas gap junctions could be identified within the comb plates as previously reported(15) in our SBFSEM data as well as TEM micrographs, we found no evidence of similar structures between neurites of nerve net neurons that would suggest the presence of electrical synapses. Additionally, a recent characterization of the complete set of *M. leidyi* innexins - responsible for the formation of gap junctions in invertebrates - did not show any mRNA expression in situ hybridization experiments in nerve net cell bodies(23). We did however observe synaptic triads and plasma membrane contacts of unknown molecular structure that connect the SNN externally to polster and mesogleal neurons.

Previous characterizations of ctenophore nerve nets have been predominantly based on traditional histochemical staining techniques(9, 24), and more recently on fluorescence microscopy of antibody staining against alpha-tubulin(10, 12, 13, 25). Although both techniques provide valuable insight into the general organization and location of ctenophore neurons, they do not allow investigating the ultrastructure and nature of neuronal connections. Data from transmission electron microscopic serial sections(26, 27) may also have overlooked this special syncytial architecture due to the difficulty to produce continuous section series over such a large volume. Besides reports on single self-anastomosing neurites in other animals(28–30), the presence of a complete syncytial nerve net has only been reported for cnidarian, medusae-like colonial polyp *Velella*(31, 32). However, at the best of our knowledge, the syncytial organization of this nerve net has not yet been verified on an ultrastructural level. At this point in time, we found this feature only in the ctenophore *M. leidyi* nerve net but further analysis across nerve net-bearing animals may provide exciting insights into early nervous system evolution and modes of neuronal connectivity.

Although neurite fusion and pruning seem to be a common principle during the early neural development in many animals(33, 34) we do not consider the syncytial cydippid SNN to be completely remodeled by such a process later in development. It was suggested that the early cydippid-phase is not a larval but rather autonomous life history phase of *M. leidyi* and other ctenophores(6). Indeed, cydippid-phase *M. leidyi* are free-swimming pelagic predators, able to reproduce and exhibit complex behaviors as described for their second, reproductive, lobate-phase(35–37).

Our identification of the non-synaptic architecture of the cydippid-phase SNN raises the intriguing question about the mechanism of signal propagation. Genome and single cell transcriptome analyses revealed that *M. leidyi* SNN neurons express a voltage gated calcium (Ca_v_), 35 potassium (K_v_) and two non-specific sodium (Na_v_) channels(14, 38, 39). These numbers are similar to neurons of other animals and ctenophore SNN neurons are therefore potentially able to produce membrane potential or even action potentials(40). Moreover, the presence of numerous peptidergic vesicles in the SNN suggests that signal transmission also occurs through neuropeptide release, and the Ca_v_ channel expressed in these cells might be involved in exocytosis(14, 41). Therefore, we can speculate that the SNN could function as a neuroendocrine system that is able to release transmitters into the mesoglea via vesicle fusion with the plasma membrane at different neurite sites. Such a system would require only a minimum number of chemical synapses and, if acting at short distances, may reach enough effector cells. Indeed, studies on the conduction velocity in ctenophores have shown a slower speed of signal propagation compared to nerve nets and conducting epithelia of other animals(42), indicating that signal propagation could be non-synaptic.

Additionally, our ultrastructural identification of simple circuits now provides a basis that allows a better understanding of how mechanoreception, swimming and prey capture behavior in young cydippid-phase ctenophores could be facilitated. Numerous sensory neurons are connected through chemical synapses to the nerve net which in turn forms chemical synapses onto effector cells like the comb rows or ciliated groove cells(14). Type 1 ciliated sensory cells and type 4 filopodiated sensory cells, previously described as ‘Tastborsten’ and ‘Taststifte’(9), have been postulated to be sensitive to water vibrations and touch(17, 43, 44). Their abundance throughout the epidermis and direct cell-cell contact to the nerve net (many through chemical synapses) highlights the importance of localized vibration and touch information to be transmitted directly to the SNN. Morphological analysis allows us to speculate that a type 2 sensory cell, which wraps around polster cells, may be able to detect water flow and thus alter comb beat frequency whereas a type 3 sensory cell, whose multiple cilia are in close contact to the tentacle, may be triggered by food capture. Functional experiments are needed to fully understand the activity of these circuits and unravel the different modes of signal transmission utilized by the different ctenophore neuronal cell types. This study is limited to the analysis of an early developmental stage where fixation of whole animals with high pressure freezing is still possible. Comparison to other ctenophore species and investigation of later life history stages of *M. leidyi* is needed to clarify if a syncytial SNN is a feature restricted to an early ontogenetic phase in only a few species or if it is a common feature of all ctenophores. This approach will also provide valuable insights into the development of the syncytial SNN: do neurons divide, but remain connected in the cydippid SNN or do neurites from different cell bodies reach out and fuse?

Whether neurons of animals have a single origin or possibly originated more than once during evolution is a debated topic. The existing data on the ctenophore nervous system show a unique mosaic of cellular and syncytial components with distinct evolutionary histories. It will be a major future challenge to clearly identify the novel parts of the mosaic that may have evolved independently and the pre-existing parts that where strongly modified, possibly even beyond recognition. Our study highlights that the resemblance between the nerve net of ctenophores and the nerve nets of cnidarians and bilaterians might only be superficial, as it appears that their connectivity is fundamental different. Our ultrastructural analysis of the ctenophore SNN not only puts ctenophores at the center of nervous system evolution, but also provides a unique opportunity to explore the boundaries of nervous system organization and function.

## Supplementary Material

Movie S1

Supplemental material

## Data Availability

All data are available in the manuscript or the supplementary material.
