# Supplementary material for: Syncytial nerve net in a ctenophore adds insights on the evolution of nervous systems
Source: Science. Author manuscript; Available in PMC 2025 Apr 5. (PMC7617566; doi:10.1126/science.ade5645)
Supplement: Supplemental material [file EMS204032-supplement-Supplemental_material.docx]

Supplementary Materials for

**Syncytial nerve net in a ctenophore sheds new light on the early evolution of nervous systems**

Pawel Burkhardt^1^, Jeffrey Colgren^1*^, Astrid Medhus^1*^, Leonid Digel^1^, Benjamin Naumann^2^, Joan J Soto-Àngel^1^, Eva-Lena Nordmann^1^, Maria Y Sachkova^1^, Maike Kittelmann^3^

Correspondence to: [pawel.burkhardt@uib.no](mailto:pawel.burkhardt@uib.no) & [maike.kittelmann@brookes.ac.uk](mailto:maike.kittelmann@brookes.ac.uk)

*These authors contributed equally to this work

**This PDF file includes:**

Materials and Methods

Figs. S1 to S7

Table S1

Captions for Movie S1

**Other Supplementary Materials for this manuscript include the following:**

Movie S1

Materials and Methods

Animal husbandry

For electron microscopy experiments, *M. leidyi* cydippids were 1-day old (i.e., < 24 hours after hatching or < 48 hours post fertilization). Animals were obtained from two months old, 3-5 mm cydippids as previously described(*14*). Briefly, the parental generation was kept in 300 mL beakers with 5-10 individuals per beaker in sea water at 20-22 °C, 27 ppt and pH 7.9 – 8.1. Ctenophores were fed with living *Brachionus* (rotifers) once a day and five times a week with a final density of 10 prey/mL. Beakers were washed every five days by carefully transferring the cydippids into beakers with new seawater. Seawater used for both the ctenophore and the rotifer culture was first filtered through a combination of 10, 5 and 1 µm mechanical filters, activated charcoal and UV irradiation. *Brachionus* were kept in 6 L transparent buckets and fed with 8-10 mL of commercial concentrated microalgae RGcomplete™ distributed in 2-3 dose/day and five times a week. In the described conditions, cydippids become reproductive ca. one week after hatching and spawn daily and continuously for years. Hatching occurs 22-26 hours after spawning and fertilization.

Immunohistochemistry

The mature neuropeptide deriving from the ML02736a precursor was predicted earlier(*14*). The peptide was chemically synthesized with the addition of an extra Cys residue at the N-terminus (ML02736a, VYKGYNGGNRVWYamide) to enable conjugation to KLH and used for immunization of rabbits followed by affinity purification by Genscript.

Animals were fixed 3-4 days post fertilization in ~ 16% Rain-X® in artificial sea water (ASW) for 1hr at room temperature (RT), followed by further fixation in ice cold 3.7% formaldehyde in ASW for 1hr on ice(*46*). Cydippids were washed four times in PTW buffer (1.8 mM KH_2_PO_4_, 10 mM Na_2_HPO_4_, 0.137 M NaCl, 2.7 mM KCl, 0.1% Tween-20, pH 7.4) or until no Rain-X® droplets were observed. The cydippids were stored in PTW at 4°C up to a week. Animals stored for longer were dehydrated trough a methanol series in PTW [50%, 75%, 100% (v/v)] and kept at -20°C and subsequently rehydrated trough methanol in PTW series [60%, 30% and 0% (v/v)] before use. Animals to undergo immunostaining were washed five times for 5 minutes in PBTx (0.2% Triton X100 in PBS (137 mM NaCl, 2.68 mM KCl, 10.14 mM Na_2_HPO_4_, 1.76 mM KH_2_PO_4_, pH 7.4)), before blocking with 1% Bovine serum albumin in PBTx for 1hr at RT. ML02736a antibody was combined with mouse E7 beta tubulin antibody (DSHB). The primary antibodies were diluted 1:100 in blocking solution, spun down at 16000 rcf for 10 minutes, and the supernatant was used for overnight incubation at 4°C. Samples were subsequently washed six times in PBTx for 15 minutes at RT, before secondary antibodies, goat anti-rabbit 647 (ab150083) and goat anti-mouse 488 (ab15017), were diluted 1:250 in blocking solution, spun down at 16000 rcf for 10 min and the supernatant was used to stain the animals overnight 4°C. The samples were washed three times in PBTx for 15 min followed by five 5-minute washes in PBS and mounted in Vectashield antifade mounting medium containing DAPI (Vector Laboratories). Samples were imaged on an Olympus FV3000 Confocal Laser Scanning Microscope and processed in Imaris.

Western blot analysis

In total 10 ctenophores (0.5 cm in size and not fed for 8 h) were transferred into a 2 ml Eppendorf tube and sea water was carefully removed (the total wet weight after sea water removal was 650 mg). Ctenophores were resuspended in 1.5 ml ice cold lysis buffer (20 mM potassium phosphate buffer, pH 7.4, 150 mM NaCl, 1 mM EDTA, 1 mM EGTA, 1% Triton X-100) containing protease inhibitor cocktail (Roche) and centrifuged for 10 min at 1°C and 13 000× g. The lysate (2 mg or 6.5 mg based on wet weight pellet) was loaded onto a 16.5% Tris-Tricine Gel and electrophoresis was performed at room temperature at 80 mV. The separated proteins were blotted onto a 0.2 μm PVDF membrane and blocked for 1 h at room temperature on a shaker in blocking buffer (3% BSA in PBS containing 0.2% Tween 20). The PVDF membrane was incubated with anti-ML02736a antibody (1:500) for 1 h at room temperature in blocking buffer. The membrane was washed extensively in PBS containing 0.2% Tween 20 and stained with secondary antibody goat anti-rabbit IgG, horseradish peroxidase conjugate (ab97051; abcam) in blocking buffer (1:10 000) for 1 h at room temperature. The membrane was washed again in PBS containing 0.2% Tween 20 and the staining was visualized using the Clarity Max Western ECL Substrate (BioRad).

TEM and SBFSEM sample preparation and imaging

1-day old *M. leidyi* cydippids were fixed and imaged using High Pressure Freezing and Freeze substitution as previously described(*14*). Briefly, *M. leidyi* cydippids were frozen in 20% BSA in seawater (Baltech HPM010), freeze substituted with a mix of 1% UA and 1% Osmium in acetone over 72 hours from -90 to 4 °C. Additional en block staining with 1% tannic acid in acetone for 2 hrs and subsequently with 1% osmium in acetone for 1 hr was performed at room temperature. Ctenophores were then infiltrated and embedded in 812 Epoxy resin and cured at 60 °C for 30 hrs.

SBFSEM images were collected with a Merlin Compact SEM (Zeiss, Cambridge, UK) with the Gatan 3View system and Gatan OnPoint BSD with pixel size 5 nm, dwell time 1us, 20 nm Aperture, 1.8 kV acceleration voltage in high vacuum with Zeiss FocalCC set to 100%. Section thickness was 100 nm.

Series of 50 nm thin sections for TEM were collected with PowerTome ultramicrotome (RMC) and imaged with a Jeol JEM-1400Flash with a Gatan OneView 16 Megapixel camera at 120kV.

SBFSEM and TEM datasets have been made available under <https://zenodo.org/record/7278449#.Y3IcynbMJaR>

3D reconstruction

The dataset was binned in X and Y to a resolution of 30x30x100 nm voxel size. 3D reconstruction was performed as previously described(*14*). Briefly, to reconstruct a *M. leidyi* whole mount cydippid SBFSEM sections were imported as z stacks into the Fiji(*47*) plugin TrakEM2(*48*) and automatically aligned using default parameters. Alignments were manually curated and adjusted if deemed unsatisfactory. Whole cells (SNN, comb cells, mesogleal neurons, sensory cells), tentacle and organelles were manually segmented, and 3D reconstructed by automatically merging traced features. Meshes were then smoothed in TrakEM2. For the generation of the 3D animation the .obj file from TrakEM2 was exported and imported into Blender 3.0.1(*49*). The membranes of the SNN were rendered transparent to reveal the underlying dense core vesicles. No other transformations that could affect the cells’ morphology have been performed. The entire EM reconstruction was animated using the basic suite of 3D animation functions of the software at 24 frames per second.

Microinjection of fluorescent dye into embryos

2 cell stage embryos were injected with fluorescent membrane stain DiI (DiIC18(3); ThermoFisher L7781A). Microinjections were done similarly to the method described by Jokura et al 2019(*50*). DiI was prepared as described in Martindale & Henry 1995(*51*). Microinjection needles were backfilled with dye dissolved in soybean oil (5mg/mL) and a droplet (Suppl. Fig 2D) was injected into the cytoplasm of one of the cells. Embryos were left to develop in dark at 20 ̊C for 2-3 days and imaged live by confocal microscopy using an Olympus FV3000RS microscope. We noted that proper development following injection presents a significant bottleneck in that only ~10-20% of individuals are usable (in comparison to >80% of individuals develop properly without injection). A total number of 31 individual ctenophore eggs were injected, 6 of them developed normally and we were able to image 5 of them (one showed very limited general signal intensity). All of the 5 imaged animals showed DiI signal in the non-injection side.


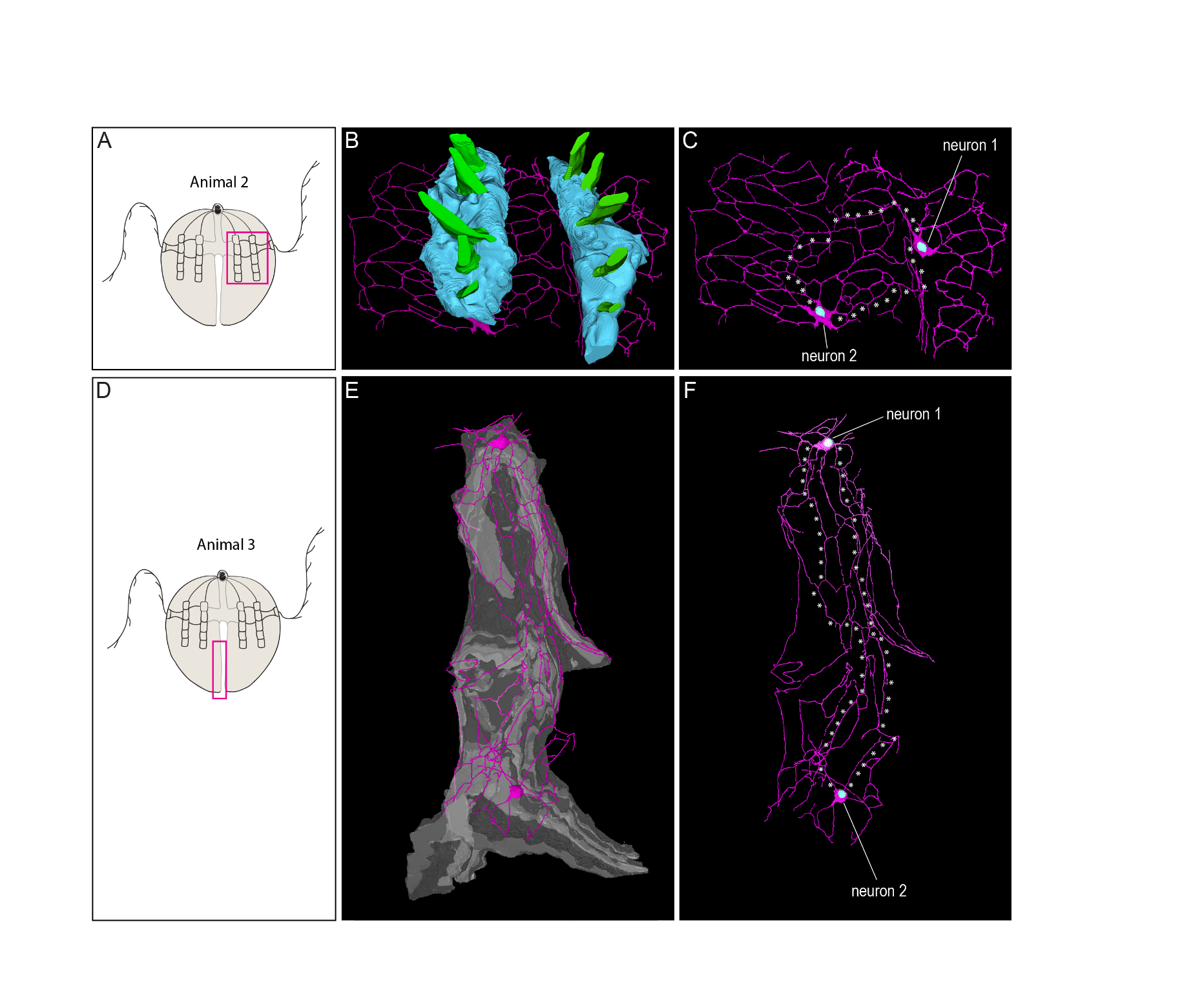
Fig. S1. Connectivity and ultrastructure of the ctenophore SNN beneath two comb rows and along the gut in two additional cydippid individuals.

**(A)** Localization of imaged and reconstructed area is indicated with a pink square. **(B)** 3D reconstruction of the nerve net and comb rows from SBFSEM data of a 1-day old cydippid. **(C)** White asterisks indicate examples of continues membrane between cell bodies of neuron 1 and 2. **(D)** Localization of imaged and reconstructed area is indicated with a pink square. **(E)** 3D reconstruction of the nerve net and gut surface from SBFSEM data of a 1-day old cydippid. **(F)** White asterisks indicate examples of continues membrane between cell bodies of neuron 1 and 2.


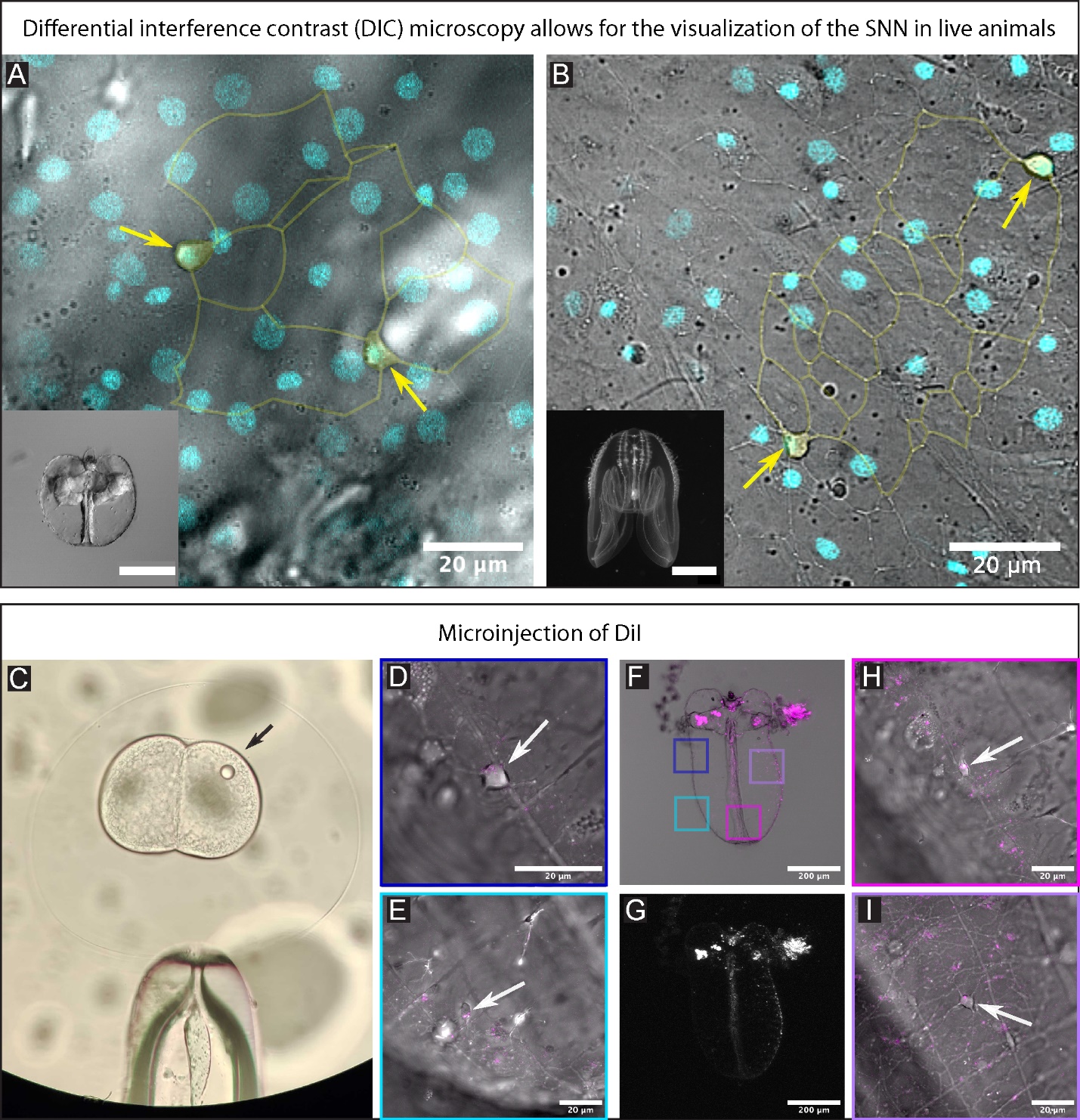


**Fig. S2. SNN architecture persists past cydippid stage and three-day old cydippid injected with DiI at 2-cell stage. (A)** DIC images of epidermis of live two-day old cydippid with two SNN cell bodies and aligned neurites highlighted in yellow (DNA shown in cyan). Individual imaged is shown in the bottom left (scale bar 200µm). **(B)** Image taken from a lobed adult, showing similar neurite branching pattern and multiple pathways between cell bodies (highlighted in yellow, DNA in cyan). Inset image shows a similar sized animal from with the image was acquired (scale bar 4mm). **(C)** Injection into two-cell staged embryo. Oil droplet containing dye is visible in one cell (black arrow). **(D-I­)** Images acquired at 3 days post injection. **(F&G)** DiI labeling of roughly half the cydippid (magenta in F and gray in G), with only one of the two tentacles showing signal. **(D&E)** Intracellular DiI signal (magenta) over DIC images of SNN cells (white arrows; identified by connection to neurites), located in the body wall opposite the stained tentacle (blue and cyan boxes in F). **(H&I)** Two examples of DiI signal (magenta) within SNN cells located on the side of the body with the labeled tentacle (magenta and purple boxes in F). All SNN cells that were observed in the injected individual showed intracellular DiI signal within the cell body, regardless of location in the animal.


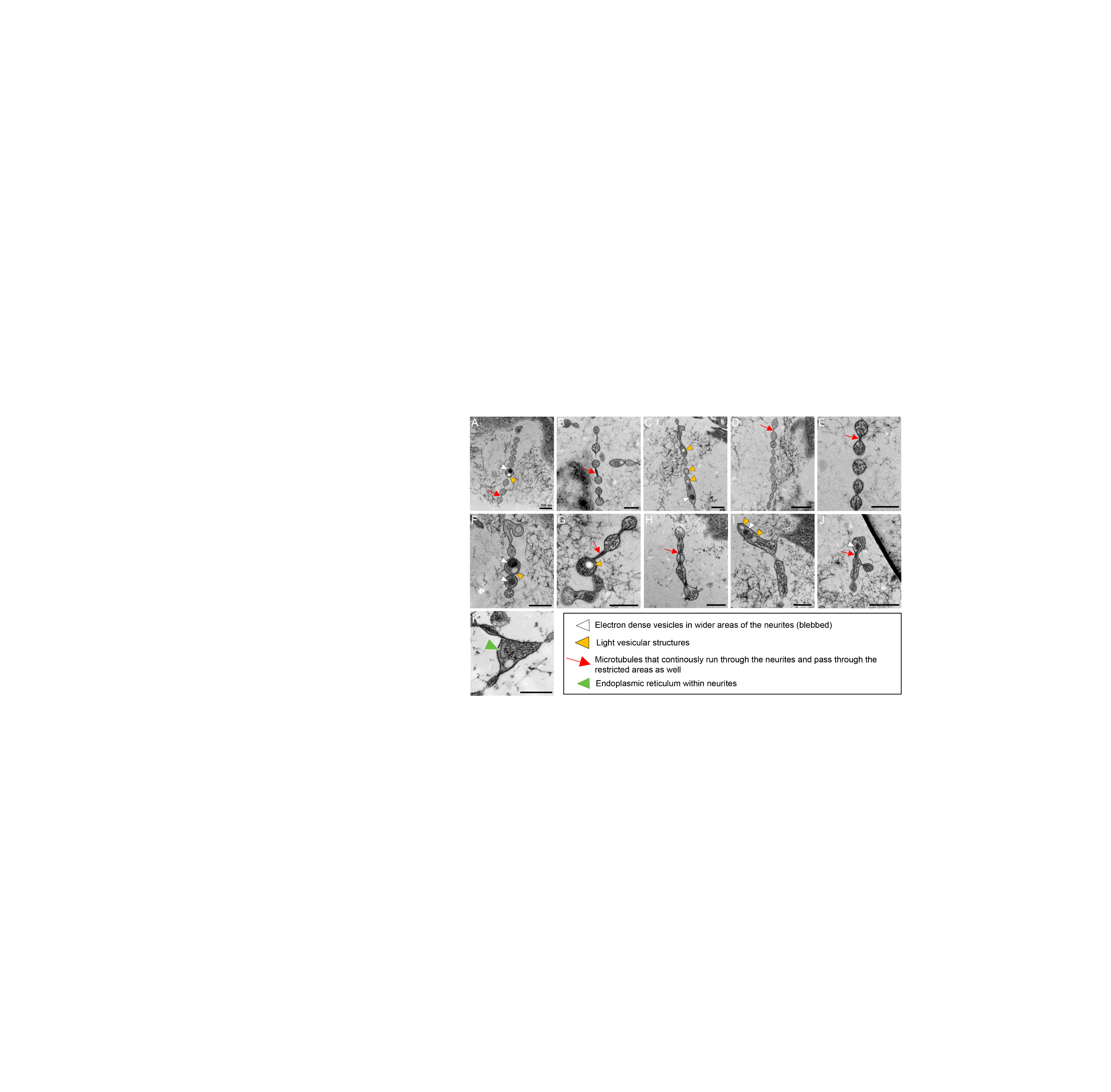
 Fig. S3. Blebbed neurite morphology of ctenophore SNN neurons.

**(A-K)** High resolution TEM micrographs of the unique structure of *M. leidyi* SNN neurites. Scale bar in all panels: 500 nm


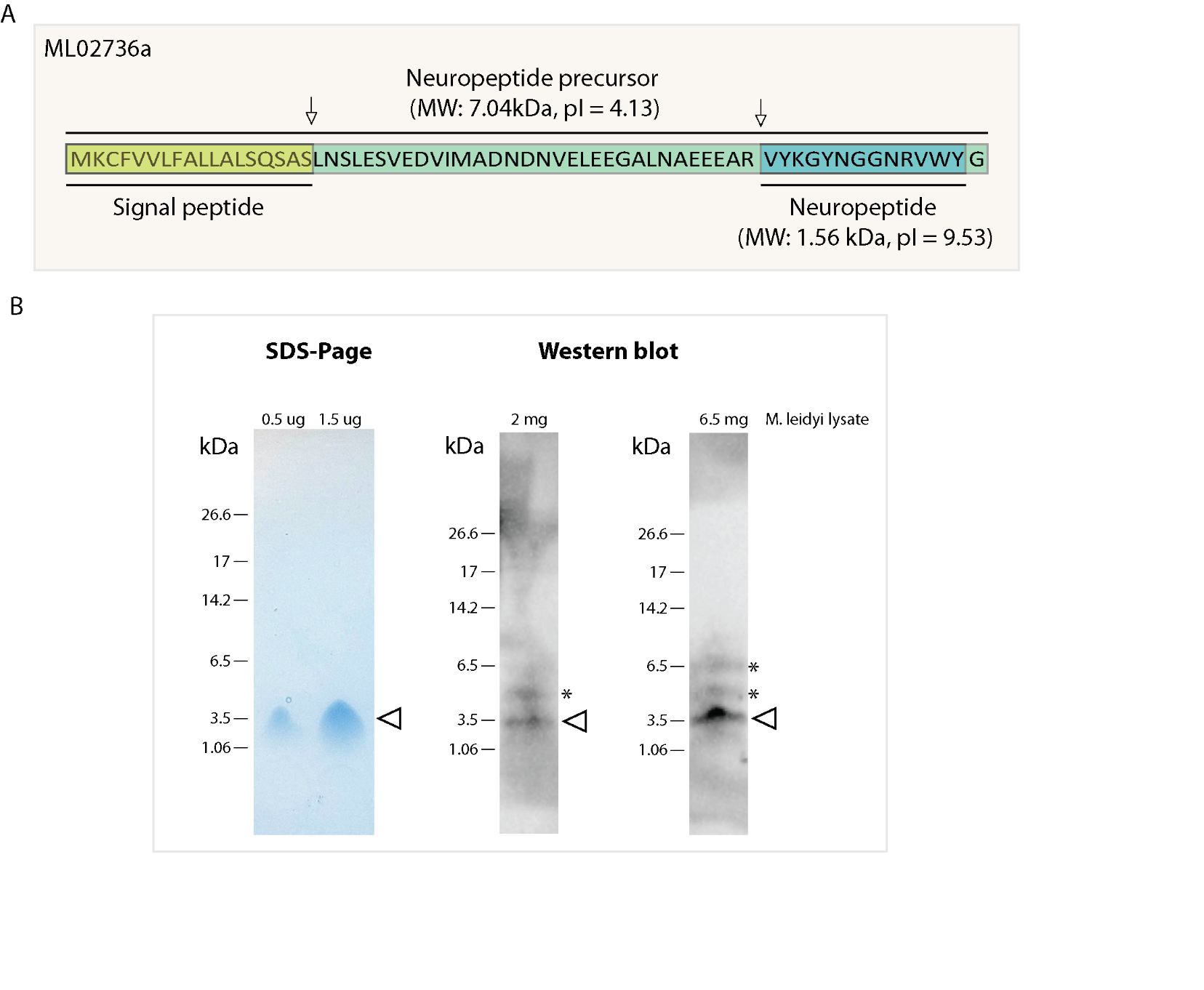


Fig. S4. Detection of ML02736a expression using western blotting analysis

**(A)** Protein sequence of the neuropeptide precursor ML02736a (light green) and predicted mature neuropeptide (light blue) with molecular weight (MW) and isoelectric point (pI) indicated. The two predicted cleaving sites are indicated with arrows. **(B)** Left: 16.5% Tris-Tricine SDS-Page allows for the visualization of the synthesized mature neuropeptide at 3.5 kDa (white arrowhead). The difference between the calculated MW of 1.56 kDa versus the observed MW of 3.5 kDa can be explained by the high pI of 9.53 of the mature neuropeptide and overall small MW. Right: *M. leidyi* lysates run on a 16.5% Tris-Tricine gel, transferred onto a PVDF membrane, and probed with antibodies against ML02736a. The antibody recognizes a strong band of approximately 3.5 kDa (white arrowheads). Note the very similar running behavior compared with the synthesized mature neuropeptide. The antibody also recognizes 1-2 additional, much weaker bands of approximately 6.5 and 5 kDa (asterisks). The observed MW of 6.5 kDa is very close to the calculated MW of 7.04 kDa and indicates, that the ML02736a neuropeptide precursor is also detected. The observed MW of 5 kDa is very close to the calculated MW of 5.1 kDa and indicates, that the neuropeptide precursor without the signal peptide is detected.


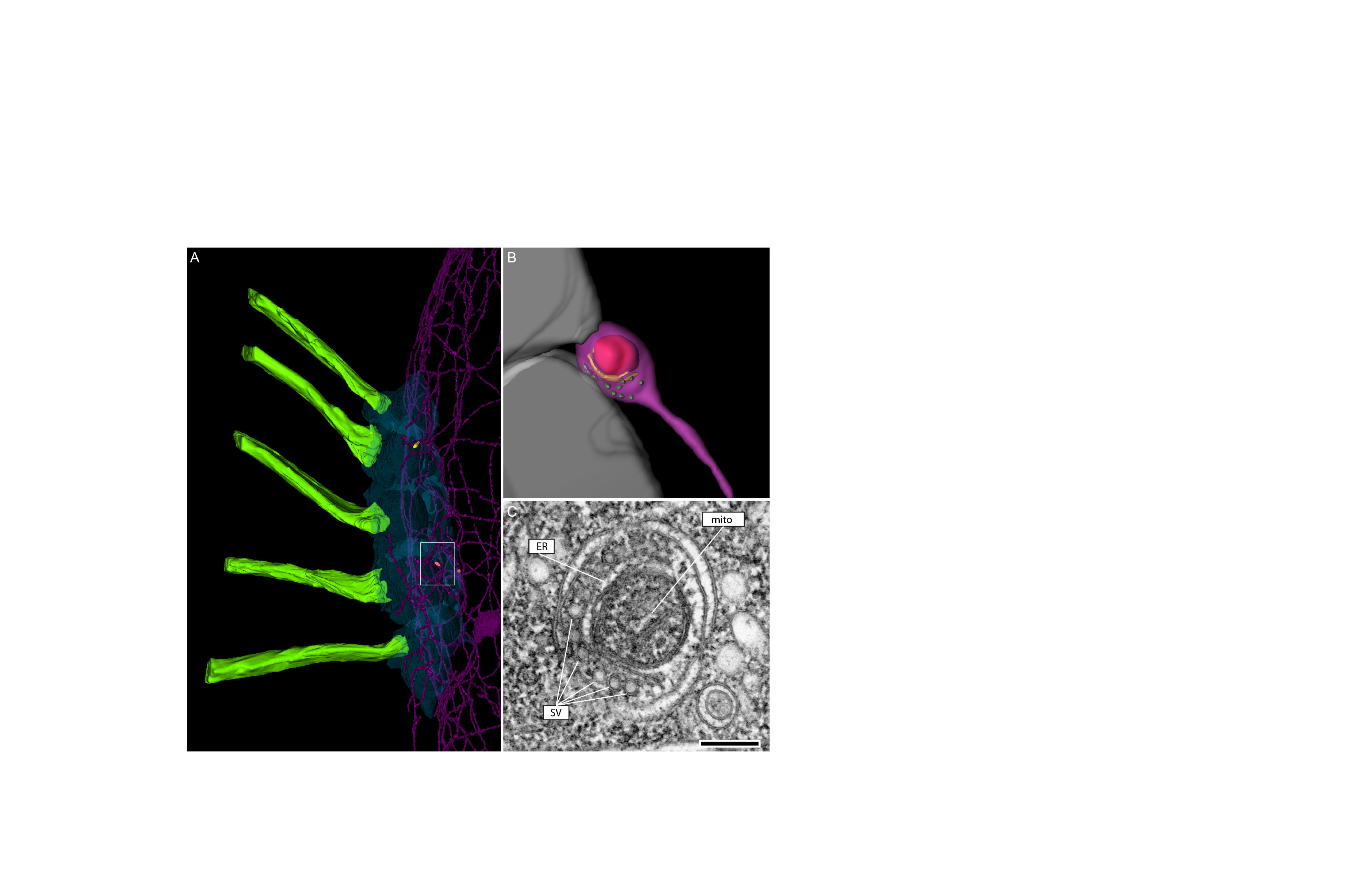
Fig. S5. SNN neuron synapses onto comb cells.

(A) 3D reconstruction of comb row, SNN neurons and 3 synapses (yellow) from SBFSEM data. White box: synapse shown in B. (B) SBFSEM data 3D reconstruction of SNN neuron chemical synapse onto comb cell. Red: mitochondria; yellow: endoplasmic reticulum; green: synaptic vesicles; grey: comb cells. (C) High resolution TEM micrograph of a chemical synapse contacting comb cells. Note the typical presynaptic triad of mitochondrion (mito), ER (endoplasmic reticulum) and SVs (synaptic vesicles). Scale bar: 200 nm


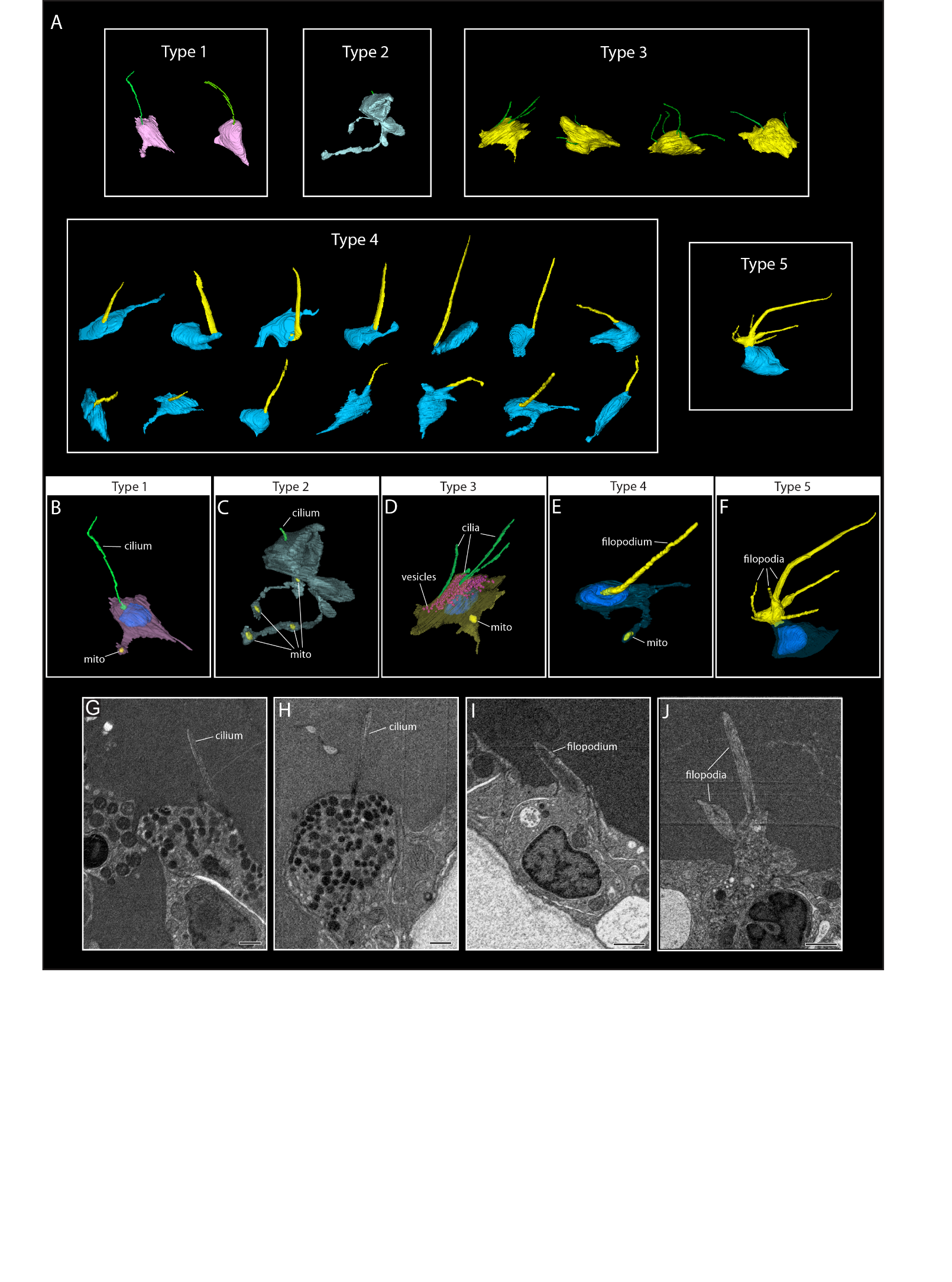
**Fig. S6.** **Multiple sensory cells on the epidermis of the ctenophore *M.* *leidyi*.**

(A) 3D reconstructed sensory cells were grouped into 5 types based and their cellular protrusions. (B-F) Representative sensory cell of each type (1-5) including some of the intracellular organelles. (G, H) Two sensory cells with cilia. (I, J). Two sensory cells with filopodia. Scale bars G, H = 500 nm; I, J = 1 um.


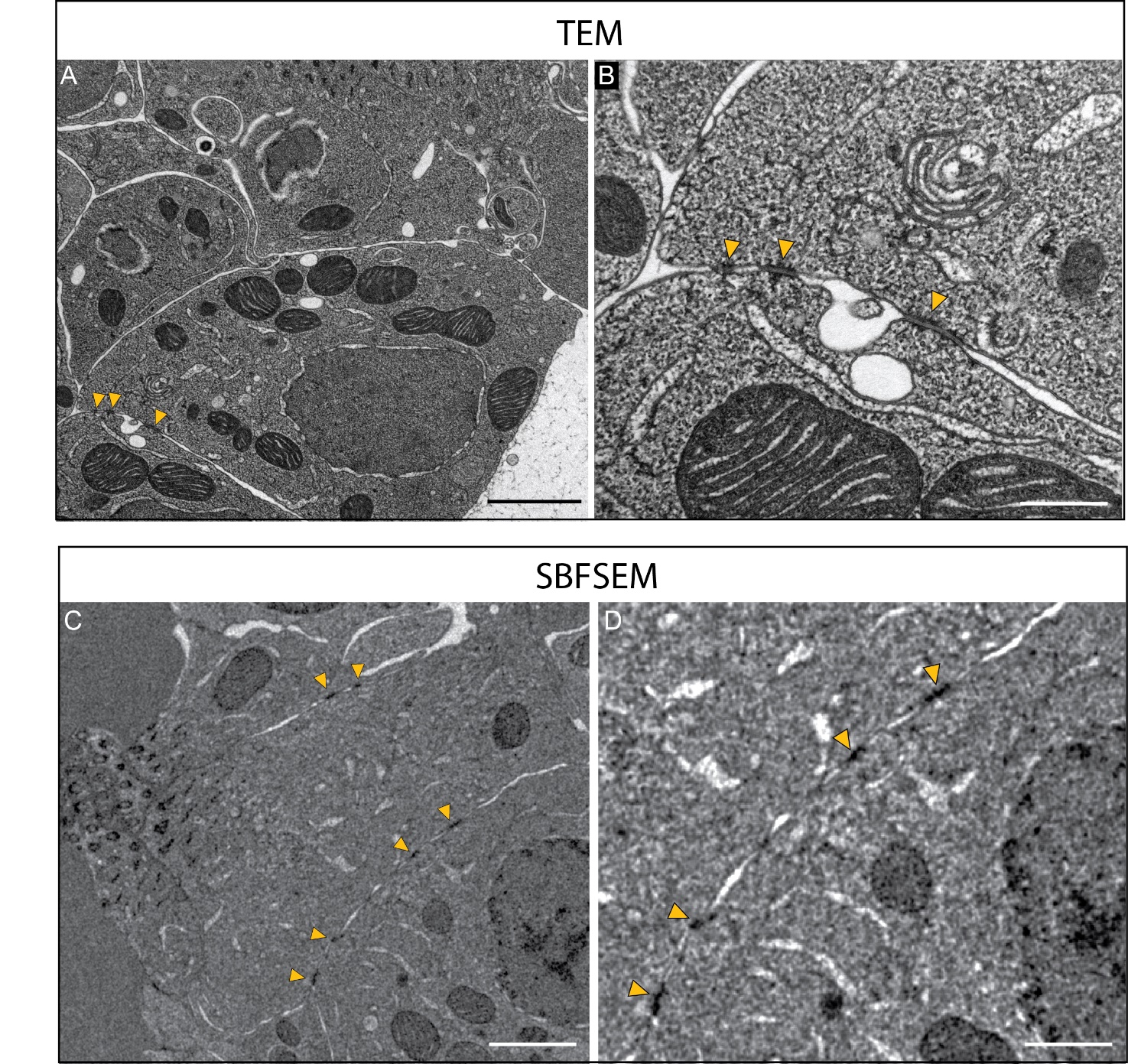
Fig. S7. Gap junctions between comb plate cells. Gap junctions are visible as electron dense patches with TEM (A and B) as well as SBFSEM (C and D) imaging techniques. Scale bars A, C = 2µm, B, D = 500nm

| **Table S1. Sensory cell types detected in *M. leidyi* 1-day old cydippid** | | |
| --- | --- | --- |
| **Sensory cell type** | **Characteristic traits** | **Synapse** |
| Type 1 | single long cilium with onion root basal body | yes* |
| Type 2 | single short cilium, no onion root basal body, long neurites | yes* |
| Type 3 | multiple cilia, no onion root basal body, large dense core vesicles underlying cilia | yes* |
| Type 4 | single filopodium | yes* |
| Type 5 | multiple filopodia | no |

*detected in some

Movie S1. 3D reconstruction of the SNN, comb rows, sensory cells, mesogleal neurons and a tentacle from SBFSEM data of a 1-day old cydippid
